# Pharmacological Treatment of Depression in Alzheimer’s Disease: A Challenging Task

**DOI:** 10.3389/fphar.2019.01067

**Published:** 2019-09-27

**Authors:** Tommaso Cassano, Silvio Calcagnini, Antonio Carbone, Vidyasagar Naik Bukke, Stanislaw Orkisz, Rosanna Villani, Adele Romano, Carlo Avolio, Silvana Gaetani

**Affiliations:** ^1^Department of Clinical and Experimental Medicine, University of Foggia, Foggia, Italy; ^2^Department of Physiology and Pharmacology “V. Erspamer”, Sapienza University of Rome, Rome, Italy; ^3^Morphological Science Department of Human Anatomy, Medical Faculty, University of Rzeszów, Rzeszów, Poland; ^4^Department of Medical and Surgical Sciences, University of Foggia, Foggia, Italy

**Keywords:** Alzheimer’s disease, depression, amyloid-β peptide, tricyclic antidepressants, selective serotonin reuptake inhibitors, serotonin

## Abstract

Besides the memory impairment, Alzheimer’s disease (AD) is often complicated by neuropsychiatric symptoms also known as behavioral and psychological symptoms of dementia, which occur in one-third of patients at an early stage of the disease. Although the relationship between depressive disorders and AD is debated, the question if depression is a prodromal symptom preceding cognitive deficits or an independent risk factor for AD is still unclear. Moreover, there is growing evidence reporting that conventional antidepressants are not effective in depression associated with AD and, therefore, there is an urgent need to understand the neurobiological mechanism underlying the resistance to the antidepressants. Another important question that remains to be addressed is whether the antidepressant treatment is able to modulate the levels of amyloid-β peptide (Aβ), which is a key pathological hallmark in AD. The present review summarizes the present knowledge on the link between depression and AD with a focus on the resistance of antidepressant therapies in AD patients. Finally, we have briefly outlined the preclinical and clinical evidences behind the possible mechanisms by which antidepressants modulate Aβ pathology. To our opinion, understanding the cellular processes that regulate Aβ levels may provide greater insight into the disease pathogenesis and might be helpful in designing novel selective and effective therapy against depression in AD.

## Introduction

Alzheimer’s disease (AD) is the most common type of dementia in western countries, corresponding to about 60% of the cases while vascular dementia is the second, with 20% of all the cases (Kalaria et al., 2008; Rizzi et al., 2014). According to World Alzheimer’s report 2018, 50 million people worldwide are living with dementia, and this number is projected to increase to more than 150 million by 2050 (https://www.alz.co.uk/research/WorldAlzheimerReport2018).

Memory dysfunction is a symptomatic feature of AD, which is characterized pathologically by amyloid-β peptide (Aβ) deposition and neurofibrillary tangles (NFTs) (Querfurth and LaFerla, 2010; Tramutola et al., 2018; Sharma et al., 2019).

Besides the memory impairment, AD is often complicated by neuropsychiatric symptoms also known as behavioral and psychological symptoms of dementia (BPSD), which occur in one-third of patients at an early stage of the disease (Assal and Cummings, 2002). In particular, BPSD in AD patients include among others, hallucination, sleep disorder, depression and anxiety, appetite disorder, and hyperactivity (Aalten et al., 2007; Bellanti et al., 2017). This comorbidity complicates diagnosis, influences treatment strategies, outcomes and finally quality of life, and affects individuals and caregivers (Modrego, 2010).

Studies from clinical settings have suggested that the prevalence of a “major depressive episode” in AD patients is 20–25%, with other depressive syndromes, including minor depression affecting an additional 20–30% of patients (Pearlson et al., 1990; Assal and Cummings, 2002; Shim and Yang, 2006; Richard et al., 2013; Leong, 2014). AD patients with major depression show a greater and faster cognitive impairment compared to non-depressed patients (Milwain and Nagy, 2005) and, surprisingly, neuritic plaques and NFTs are more evident in the cerebral parenchyma of AD patients with comorbid depression than non-depressed AD patients (Rapp et al., 2008).

Although the link between depressive disorder and AD is debated, the question whether depression is a prodromal symptom or an independent risk factor for AD still remains unresolved. Moreover, there is growing evidence in the literature that conventional antidepressants are not effective in depression accompanied by AD (Pomara and Sidtis, 2007) and, therefore, there is an urgent need to understand the neurobiological mechanism underlying the resistance to the antidepressants.

Hence, we examined the possible link between depression and AD, and we focused on the resistance of antidepressant therapies in dementia. Subsequently, we explored probable mechanisms by which antidepressants modulate Aβ pathology. The latter evidence may be helpful in designing novel selective and effective therapy against depression in AD.

## Relationship Between Depression and AD: Prodromal Symptom or Risk Factor?

To date, converging evidence suggests that depression may represent a risk factor for the development of AD (Steffens et al., 1997; Modrego and Ferrández, 2004; Bartolini et al., 2005; Ownby et al., 2006), mostly when depressive symptoms occur more than 10 years before the onset of AD (Speck et al., 1995). To this regard, a systematic meta-analysis study evaluated whether observed risk for developing AD was related to the interval between diagnosis of depression and AD (Ownby et al., 2006). The authors found that there was a positive correlation between this interval and the risk of developing AD, suggesting that, rather than a prodrome, depression can be considered as a risk factor for AD. This study reported that patients with a history of depression were more prone to be affected by AD later in life (Ownby et al., 2006).

**Figure 1:**
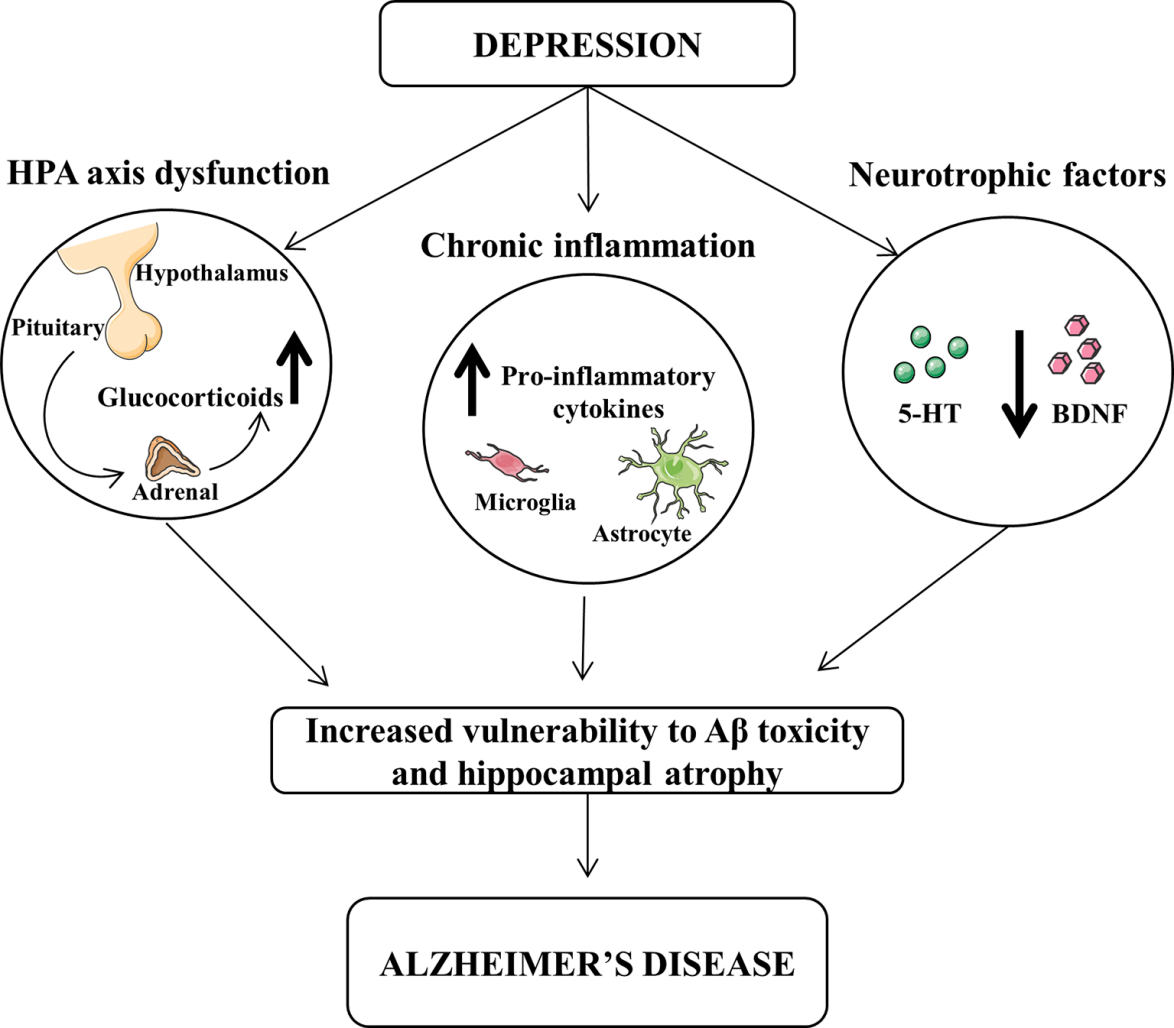
Dysfunction of hypothalamic-pituitary-adrenal (HPA) and decrease of neurotrophic factors and chronic inflammation exert a central role in the pathophysiology of both depression and Alzheimer’s disease (AD). The increased levels of glucocorticoids and pro-inflammatory cytokines, as well as the reduced levels of brain-derived neurotrophic factor (BDNF) and serotonin (5-HT) may lead to an increased vulnerability of β-amyloid toxicity and hippocampal atrophy, thus favoring the progression from depression to AD.

Several hypotheses could be proposed for this interpretation and summarized in **Figure 1**. High cortisol levels or hypothalamic–pituitary–adrenal (HPA) axis dysregulation has been associated to depression symptoms and alteration of learning and memory (Swaab et al., 2005). Corticosteroid receptors are particularly abundant in the hippocampus, which is one of the main brain regions affected by AD pathology, and their excessive stimulation may be detrimental leading to neuronal death through an apoptotic mechanism (Sapolsky, 2000; Lee et al., 2002).

It has been demonstrated that pro-inflammatory cytokines play an important role in the expression of the clinical symptoms of depression in AD patients. In particular, tumor necrosis factor-α (TNF-α), interleukin-1β (IL-1β), and IL-6 are increased during depression, as well as associated with cognitive impairment (Parissis et al., 2004; Suarez et al., 2004; Wuwongse et al., 2010).

Moreover, neurotrophic factors have a protective action against toxic agents, which may induce neurodegeneration. Chronic treatment with antidepressants up-regulate the expression of neurotrophic factors increasing hippocampal neurogenesis (Mattson, 2004).

The elevation of cortisol and pro-inflammatory cytokines, as well as the reduction of neurotrophic factors, may lead to alterations in the monoaminergic neurotransmitters, which are downregulated in either depression or AD (Reinikainen et al., 1990; Romano et al., 2014). To this regard, it has been demonstrated that depressed patients affected by AD show neuronal degeneration in locus coeruleus and raphe nuclei, both brain regions implicated in depression (Zubenko et al., 1990). Preclinical and clinical studies have demonstrated that reduced levels of brain serotonin (5-HT) and norepinephrine occur in depression, and antidepressants lead to an increase of both neurotransmitter activities in the brain (Chen and Skolnick, 2007). Yet, similar neurotransmitter alterations are also present in AD, as we have also demonstrated in an animal model of AD (Reinikainen et al., 1990; Romano et al., 2014).

Although many treatment options are available, several clinical trials suggest that antidepressants for the treatment of depression in AD patients appear ineffective (Pomara and Sidtis, 2007; Sepehry et al., 2012). To this regard, the results of the completed United States NIMH-funded, large-scale STAR*D effectiveness trial reported a remission rate of only 70% after 12 months with up to four treatment steps (Insel and Wang, 2009). In general, the tricyclic antidepressants (TCA) are less prescribed to AD patients with depression due to their serious cardiac and anticholinergic side effects (Raji and Brady, 2001; Banerjee et al., 2013), while the selective serotonin reuptake inhibitors (SSRIs) are the most prescribed drugs although evidence for their efficacy in this population is controversial (Nyth et al., 1992; Katonaet al., 1998).

To date, no medication has been approved by the U.S. Food and Drug Administration (FDA) for the treatment of depressive symptoms in AD. Some of the evidence found in geriatric population has demonstrated that antidepressant therapy can improve the depression after 4–6 weeks of treatment (Wilson et al., 2001). However, its efficacy and safety remain inconclusive in individuals with AD, and it can be a challenging task to evaluate the role and mechanisms of antidepressants in subjects with AD (Leong, 2014; Lozupone et al., 2018). Numerous mechanisms underlying resistance to antidepressants in patients with AD have been hypothesized (for review, see Lozupone et al., 2018). In upcoming future, for developing specific and effective therapy against depression in AD, studies investigating compounds targeting alternative signal transduction pathways are warranted.

## Antidepressant Treatments Modulate Aβ Levels

Postmortem analyses of brain patients and studies on transgenic animal models have demonstrated that the major neuropathological hallmarks of AD are the extracellular deposits of Aβ plaques abundant mainly in the cortex and hippocampus (Oakley et al., 2006; Oddo et al., 2006; LaFerla et al., 2007; Cassano et al., 2011; Gatta et al., 2016). Moreover, numerous preclinical studies have firmly demonstrated that the accumulation of intracellular Aβ precedes extracellular plaque formation (Oakley et al., 2006; Oddo et al., 2006; LaFerla et al., 2007). Indeed, it has been demonstrated that intraneuronal Aβ levels decrease as extracellular plaques accumulate.

**Figure 2:**
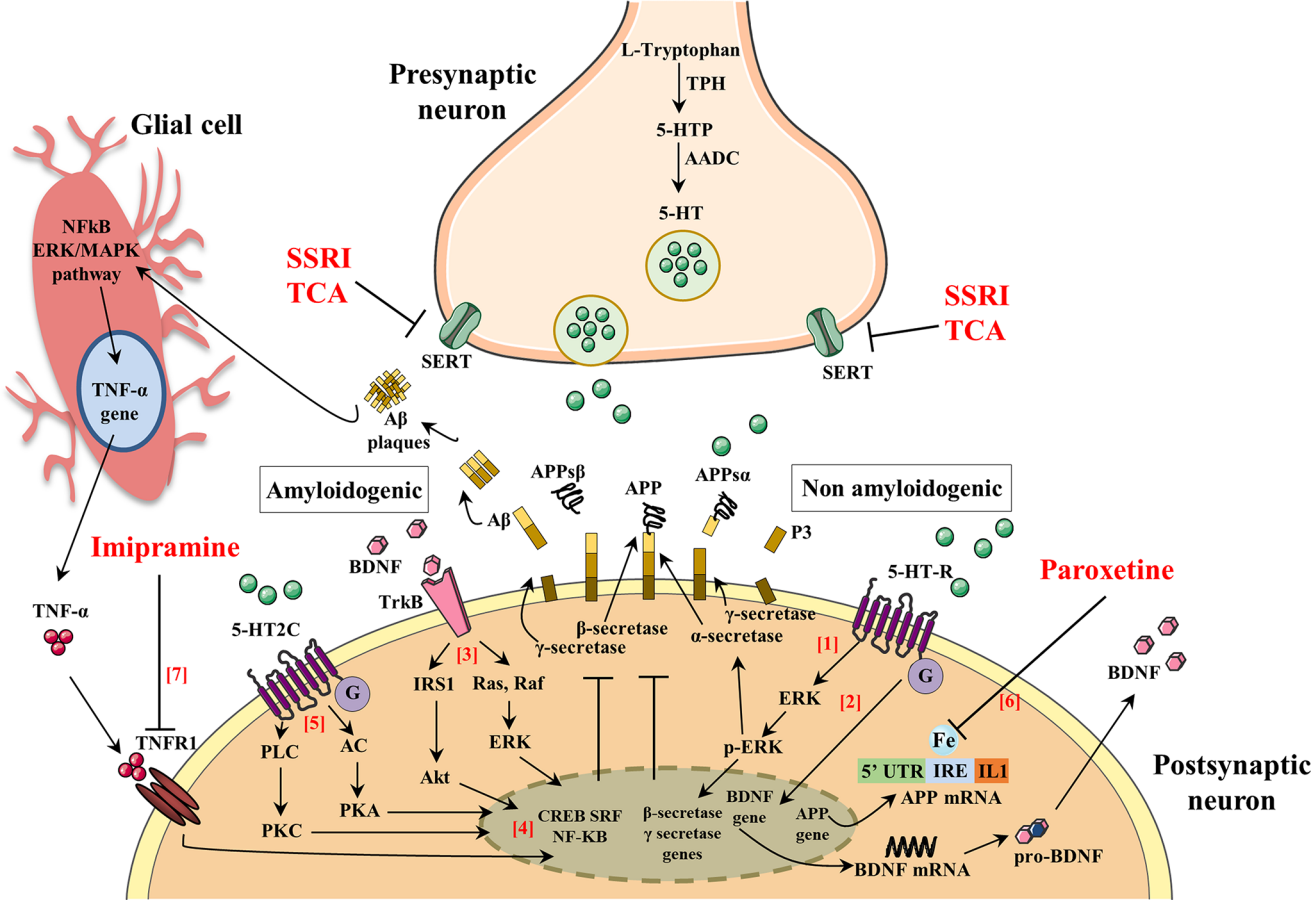
The activation of serotonin receptors (5-HT-R) initiates a signaling cascade that leads to the activation of extracellular signal-regulated kinases (ERK). Once activated, p-ERK increases α-secretase activity and reduces β- and γ-secretase cleavage of APP (1). Brain-derived neurotrophic factor (BDNF) and serotonin (5-HT) regulate synaptic plasticity, neurogenesis, and neuronal survival. The activation of 5-HT-R stimulates the expression of BDNF, which in turn enhances the growth and survival of 5-HT neurons (2). BDNF binds to its high-affinity tyrosine receptor kinase B (TrkB) resulting in the recruitment of proteins that activate two different signal transduction cascades: (i) insulin receptor substrate-1 (IRS-1), phosphatidylinositol-3-kinase (PI-3K), and protein kinase B (Akt) and (ii) Ras, Raf, and extracellular signal regulated kinases (ERK) (3). BDNF signaling pathways activate one or more transcription factors that regulate the expression of genes encoding proteins. Such transcription factors include cAMP-response-element-binding protein (CREB), serum response factor (SRF), and nuclear factor kappa B (NF-kB) that exert an inhibitory action in the amyloidogenic pathways (4). 5-HT binds 5-HT-2C resulting in the recruitment of proteins that activate two different signal transduction cascades: (i) adenylate cyclase (AC), cAMP, and protein kinase A (PKA) and (ii) phospholipase C (PLC), diacylglycerol (DAG), and protein kinase C (PKC) (5). The 5-HT signaling pathway activates one or more transcription factors that regulate expression of genes encoding proteins (CREB, SRF, and NF-kB) leading to an inhibitory action in the amyloidogenic pathways (4). The 5’-untranslated region (5’UTR) of the APP mRNA is a key regulatory sequence that determines the amount of intracellular APP holoprotein present in brain-derived cells in response to interleukin-1 (IL-1) and iron (IRE). Paroxetine acts as an intracellular iron chelator to limit the translation of APP holoprotein guided by sequences of untranslated APP mRNA 5’UTR regions (6). Tumor necrosis factor α (TNF-α) signaling, through tumor necrosis factor receptor 1 (TNFR1), mainly results in activation of the transcription factors NF-kB and induces pro-inflammatory effects that exacerbate neuroinflammation and secondary neuronal damage. Imipramine blocks TNF-α/TNFR1 signaling and prevents the appearance of cognitive deficits in AD and Aβ formation (7).

A number of pathogenic mechanisms triggering the neurodegenerative phenomena and leading to neuronal death have been described. Among them, a crucial role seems to be played by inflammation (Bronzuoli et al., 2018; Scuderi et al., 2018; Bronzuoli et al., 2019), oxidative damage (Reddy et al., 2009; Serviddio et al., 2011; Cassano et al., 2012; Cassano et al., 2016; Giudetti et al., 2018), iron deregulation (Adlard and Bush, 2006), and cholesterol metabolism (Stefani and Liguri, 2009; Barone et al., 2016). Therefore, novel therapeutical strategies aim to interfere with those pathogenic mechanisms at an early stage of the disease in order to stop/slow down the neurodegenerative process. For this reason, they have been termed “disease-modifying” drugs and should be administered to patients many years before the appearance of the first AD symptoms (Morris and Price, 2001). Consequently, if anti-Aβ therapies may be used for years or decades, then very safe compounds will likely be necessary. To this regard, SSRIs are good candidates for this purpose since their side effects are generally well tolerated, even with chronic use. In support to such hypothesis, it has been demonstrated that SSRIs reduce risk of AD in depressed individuals (Kessing et al., 2009). Therefore, individuals with a history of chronic use of antidepressant drug use may have reduced Aβ plaques and, as a result, a reduced risk of AD. Nevertheless, although this hypothesis has been postulated, it is still matter of debate whether antidepressant treatment rises or declines the level of Aβ in the brain. To this purpose, many researchers have investigated whether the activation of 5-HT receptors can regulate Aβ metabolism. **Figure 2** and **Table 1** summarize the molecular mechanisms by which antidepressant drugs may induce a reduction of the Aβ levels.

**Table 1:** Effect of antidepressant drugs on Aβ production.

PRECLINICAL STUDIES:			
Antidepressant drugs	Subjects	Effects and mechanisms involved	References
Fluoxetine, desvenlafaxine, citalopram (SSRIs)	PS1APP transgenic mice	↓ Aβ levels ↓ Aβ plaques in the cortex and hippocampus ↓ ERK signaling ↑ α-Secretase activity	Cirrito et al., 2011
Paroxetine (SSRI)	3×TgAD mice	↓ Aβ levels ↓ APP production ↓ 5-HT reuptake ↑ BDNF expression	Nelson et al., 2007 Mattson, 2004
Paroxetine (SSRI)	TgCRND8 mice	↓ Aβ levels in the cortex ↓ APP holoprotein translation driven by APP mRNA 5′ untranslated region sequences	Tucker et al., 2005 and Tucker et al., 2006
Paroxetine (SSRI)	Neuroblastoma cells (SY5Y)	↓ APP holoprotein translation driven by APP mRNA 5′ untranslated region sequences	Morse et al., 2004
Imipramine (TCA)	Rat primary basal forebrain cultures	↑ APP secretion ↑ PKC level	Pákáski et al., 2005
Citalopram (SSRI)	Rat primary basal forebrain cultures	↑ APP secretion = PKC level	Pákáski et al., 2005
Imipramine (TCA)	Swiss mice after intracerebroventricular injection of Aβ_25-35_	↓ Aβ levels in the frontal cortex ↓ TNF-α level	Chavant et al., 2010
**HUMAN STUDIES:**			
**Antidepressant drugs**	**Subjects**	**Effects**	**References**
SSRI TCA Trazodone Venlafaxine (SNRI) Bupropion Mirtazapine (NaSSA)	Plasma of elderly depressed patients	= Aβ_42_ levels ↓ Aβ_40_ levels	Sun et al., 2007
Paroxetine (SSRI) Nortriptyline (TCA)	Plasma of elderly patients with late-life major depression	= Aβ_42_ levels ↑ Aβ_42_/Aβ_40_ ratio	Pomara et al., 2006
Conventional antidepressants	Plasma of young patients affected by major depressive disorder	= Aβ levels ↑ Aβ_42_/Aβ_40_ ratio	Kita et al., 2009

SSRI, selective serotonin reuptake inhibitor; TCA, tricyclic antidepressant; SNRI, serotonin–norepinephrine reuptake inhibitor; NASSA, noradrenaline and specific serotonergic antidepressant.

Interestingly, in a preclinical study, it has been demonstrated that extracellular Aβ levels were decreased by 25% following the acute administration of several SSRI antidepressant drugs (fluoxetine, desvenlafaxine, and citalopram) and that chronic treatment with citalopram caused a 50% reduction Aβ plaques in the cortex and hippocampus of a mouse model of AD (Cirrito et al., 2011). In this study, authors demonstrated that SSRI can modulate APP processing through the activation of extracellular regulated kinase (ERK) pathway, which has been shown to suppress Aβ production *in vitro* and *in vivo* by increasing α-secretase cleavage of APP (Kim et al., 2006; Kojro et al., 2006). Once activated, ERK is able to phosphorylate specific effector proteins modulating a wide range of responses within the cytoplasm and the nucleus altering, in turn, the transcription of a wide range of genes (Kim et al., 2006; Kojro et al., 2006). Therefore, authors showed that a reduction of Aβ levels in a mouse model of AD was obtained after both acute and chronic citalopram treatments through an increase of α-secretase activity (Cirrito et al., 2011). These and many other findings suggest that the activation of 5-HT receptors by SSRI may have an “upstream” role within the amyloid cascade that may modulate the proteases involved in Aβ production itself.

Moreover, SSRI treatment showed similar effect in a triple transgenic murine model of AD (3×Tg-AD), which harbors three mutant human genes (APPswe, PS1M146V, and tauP301L) and exhibited a depressive-like phenotype with deficits of monoaminergic neurotransmissions (Oddo et al., 2003; Nelson et al., 2007; Romano et al., 2014; Scuderi et al., 2018; Barone et al., 2019; Cassano et al., 2019). Nelson and colleagues reported that pre-symptomatic treatment with the antidepressant paroxetine reduces Aβ pathology in the hippocampus and improves cognitive performance in the 3×TgAD mice (Nelson et al., 2007). In particular, 5-month-old 3×Tg-AD mice were treated for 5 months with a dose of paroxetine that inhibits 5-HT reuptake and ameliorates behavioral deficits in mouse models of anxiety and depression (Cryan et al., 2004; Goeldner et al., 2005). Taking together these results and considering the safe profile of paroxetine after chronic treatment also in elderly patients, the authors suggested that paroxetine might be a valuable therapeutic option for depressed human subjects with early AD symptoms.

The beneficial effects of paroxetine seem to be mediated by the inhibition of 5-HT reuptake resulting in both enhanced serotonergic signaling and upregulation of brain-derived neurotrophic factor (BDNF) expression (Mattson, 2004). Preclinical and clinical studies have highlighted that there is a tight interplay between SSRI treatment and BDNF expression. In particular, results from animal studies have demonstrated that paroxetine elevates both 5-HT and BDNF in huntingtin mutant mice reducing the onset and progression of the disease (Duan et al., 2004), as well as BDNF expression was increased in both hippocampus and cortex after its chronic administration in rodents (Nibuya et al., 1995; Nibuya et al., 1996). Moreover, antidepressant effects were observed when BDNF was directly injected into the rodent hippocampus (Siuciak et al., 1997; Shirayama et al., 2002). Furthermore, in human studies, it has been shown that (i) AD patients showed low levels of both 5-HT (Gottfries, 1990) and BDNF (Hock et al., 2000; Lee et al., 2005) in the hippocampus and cortex, and that (ii) antidepressants were able to increase also BDNF levels (Chen et al., 2001; Dwivedi et al., 2003).

Besides BDNF and 5-HT, antidepressant drugs may exert their effects also *via* different pathways. In fact, paroxetine seems to target APP gene expression through the 5’-untranslated region (5’UTR) of the precursor transcript suppressing translation of the APP protein (Morse et al., 2004; Tucker et al., 2005). The 5’UTR of the transcript encoding the APP (APP 5’UTR) is an important regulatory sequence that regulates the amount of intracellular APP holoprotein present in neuron cells in response to interleukin-1 (IL-1) (acute box-domain) (Rogers et al., 1999) and iron (Rogers et al., 2002). Within the 5’UTR of the APP transcript is present an Iron-responsive Element (IRE type II), which is a RNA stem loop that controls cellular iron homeostasis and is located immediately upstream of an IL-1 responsive acute box domain (Rogers et al., 2002). Therefore, the authors suggest that paroxetine may act as a chelator of intracellular iron to consequently limit APP holoprotein translation driven by APP mRNA 5’UTR sequences (Morse et al., 2004; Tucker et al., 2005). Treating 3-month-old TgCRND8 mice with paroxetine for 3 months, the authors found that paroxetine reduced the soluble and insoluble Aβ levels in the cortex of TgCRND8 transgenic mice (Tucker et al., 2005; Tucker et al., 2006). Moreover, Morse and colleagues have demonstrated that paroxetine significantly reduced the levels of APP in the neuroblastoma cells (SY5Y), whereas equivalent levels of APP-like protein 1 (APLP-1) were unchanged. As IRE sequences were absence in the 5’UTR of the APLP-1 transcript, paroxetine did not affect its levels (Morse et al., 2004).

Antidepressants seem to modulate also the expression of protein kinase C (PKC), which is a key signal transduction factor in the stimulation of APP secretion induced by the activation of 5-HT_2C_ receptors (Nitsch et al., 1996; Arjona et al., 2002). To this regard, Pákáski and colleagues investigated *in vitro* whether the TCA imipramine and the SSRI citalopram were able to modulate the PKC levels in rat primary basal forebrain cultures leading to an increased release of APP (Pákáski et al., 2005). The authors found that both imipramine and citalopram significantly increase the APP secretion (3.2- or 3.4-fold, respectively), although imipramine caused a more rapid and long-lasting APP secretion compared to citalopram. These results were accompanied by a consistent increase of PKC level after imipramine treatment while no significant effects were observed after citalopram treatment. This difference may be due to the different mechanism in monoamine uptake between antidepressants. In fact, after chronic treatment with SSRI fluoxetine, the activity of PKC was suppressed in the cortex and hippocampus of rodents (Mann et al., 1995), whereas while an increased PKC activity in rabbit and human platelets was reported after TCA administration (Morishita and Aoki, 2002). These results highlighted the primary role of antidepressants on the APP metabolism, although further investigations need to be performed in order to clarify the different profile between TCA and SSRI.

Preclinical and clinical evidences support a central role of inflammation in the pathogenesis of AD and depression (Tan et al., 1999; Dantzer et al., 2008). Human studies have demonstrated that the proinflammatory cytokines, TNF-α, and IL-1β are significantly increased in depressed subjects, as well as in the brain and plasma of AD patients (Dantzer et al., 2008). A crucial role of inflammation in the AD pathogenesis was further demonstrated by higher expression of tumor necrosis factor receptor 1 (TNFR1) in AD brain (Li et al., 2004). Moreover, the deletion of TNFR1 causes a reduction of both Aβ production and microglia activation as well as ameliorates the cognitive deficits in APP23 mice (He et al., 2007). TNF-α signaling through TNFR1 activates the transcription factors NF-κB and AP-1 and induces pro-inflammatory effects that further exacerbate neuroinflammation leading to neuronal death (Probert, 2015). Thus, the use of specific pharmacological agents that counteract TNF-α/TNFR1 signaling may be a promising therapeutic strategy to reduce the cognitive alterations and Aβ formation. To this regard, Chavant and colleagues investigated the effects of TCA imipramine on the TNF-α expression and APP metabolism using a model of Aβ_25–35_ intracerebroventricular infusion in mice (Chavant et al., 2010). Previous reports have demonstrated that intracerebroventricular injection of Aβ_25–35_ peptide induced alterations of spatial and working memory and enhanced the levels of APP and TNF-α in the frontal cortex and hippocampus of mice (Maurice et al., 1996; Lu et al., 2009). Chavant and colleagues found that imipramine prevented the Aβ_25–35_-induced deficits of both long- and short-term memories and significantly reduced the intracellular Aβ immunoreactivity in the frontal cortex counteracting the TNF-α increase induced by the Aβ_25–35_ intracerebroventricular injection (Chavant et al., 2010). Thus, these results support the claim that imipramine may be a potential candidate for the treatment of AD because of its intrinsic property to inhibit TNF-α. Overall, the preclinical studies showed a reduction of Aβ pathology after antidepressants treatment.

Differently, human studies did not show a clear-cut effect of antidepressants on Aβ metabolism. To this regard, Sun and colleagues reported that elderly depressed patients had lower plasma Aβ_42_ levels than those without depression, and such difference was not modified after antidepressant treatment with SSRI, TCA, trazodone, and all others including venlafaxine, bupropion, and mirtazapine (Sun et al., 2007). Conversely, antidepressants were able to reduce the plasma Aβ_40_ levels in depressed patients, although any significant difference was observed before treatment between subjects with depression and those without depression (Sun et al., 2007). Similarly, Pomara and colleagues confirmed that plasma Aβ_42_ levels were not affected by either paroxetine or nortriptyline, although the authors reported for the first time an elevation in plasma Aβ_42_ levels and the Aβ_42/40_ ratio in elderly patients with late-life major depression (Pomara et al., 2006). Finally, Kita and colleagues reported that Aβ_42_ was slightly increased also in young patients affected by major depressive disorder suggesting that Aβ_42_ alteration may be detrimental even in young depressed population. Moreover, the latter study confirmed that the pharmacological treatment with conventional antidepressants did not affect Aβ plasma concentrations (Kita et al., 2009).

Unfortunately, all data from clinical studies are quite mixed, and the results are difficult to interpret, due to different study methods, heterogeneous patient populations, variability in outcome measures, and concomitant treatments. Thus, more studies need to be done in order to establish whether AD patients with depression show increased or decreased plasma Aβ levels and whether these individuals may benefit from antidepressant treatments.

## Conclusion

The present review provides evidence that depression is associated with an increased risk of AD, and although many treatment options are available, several clinical trials suggest that conventional antidepressants are ineffective for the treatment of depression in AD patients. Moreover, results from human investigations do not give a clear picture on whether antidepressants are able to clearly modulate the Aβ production and eventually slow down the accumulation of the Aβ_42_ into the cerebral parenchyma. This calls for a critical analysis of the current trials on the efficacy of antidepressants, as a treatment option. In fact, although many studies have filled some of the gaps, conflicting and inconclusive results continue to represent a challenge for physicians. Moreover, depression in AD comorbidity represents a big challenge in term of correct identification and evaluation of symptoms, also because late-life depression occurs in a complex medical and psychosocial context. To this regard, the functional neuroimaging approach may contribute elucidating both the brain structure and function specifically affected in both pathologies.

This review pulls together evidence that justifies the therapeutical inefficacy of antidepressants in AD patients and promotes further research in order to design novel selective and effective therapy against depression in AD.

## Author Contributions

All authors have contributed to the writing, design and preparation of figures. The senior authors TC and SG have carried out coordination of efforts.

## Funding

This article was published with a contribution from 5 x 1000 IRPEF funds in favour of the University of Foggia, in memory of Gianluca Montel.

## Conflict of Interest Statement

The authors declare that the research was conducted in the absence of any commercial or financial relationships that could be construed as a potential conflict of interest.

The reviewer MP declared a shared affiliation, though no other collaboration, with several of the authors SC, AC, AR, SG to the handling editor.
